# Increase in Cardiac Ischemia-Reperfusion Injuries in Opa1^+/-^ Mouse Model

**DOI:** 10.1371/journal.pone.0164066

**Published:** 2016-10-10

**Authors:** Sophie Le Page, Marjorie Niro, Jérémy Fauconnier, Laura Cellier, Sophie Tamareille, Abdallah Gharib, Arnaud Chevrollier, Laurent Loufrani, Céline Grenier, Rima Kamel, Emmanuelle Sarzi, Alain Lacampagne, Michel Ovize, Daniel Henrion, Pascal Reynier, Guy Lenaers, Delphine Mirebeau-Prunier, Fabrice Prunier

**Affiliations:** 1 Institut MITOVASC, Université Angers, CHU Angers, Angers, France; 2 Laboratoire Cardioprotection Remodelage et Thrombose, Angers, France; 3 Service de Cardiologie, CHU Angers, Angers, France; 4 INSERM U1046, Université Montpellier I et II, Montpellier, France; 5 INSERM UMR 1060, CarMeN, Lyon, France; 6 INSERM UMR_S1083, CNRS UMR_C6214, BNMI, Angers, France; 7 Institut des Neurosciences de Montpellier, INSERM U1051, Université Montpellier I et II, Montpellier, France; Université catholique de Louvain, BELGIUM

## Abstract

**Background:**

Recent data suggests the involvement of mitochondrial dynamics in cardiac ischemia/reperfusion (I/R) injuries. Whilst excessive mitochondrial fission has been described as detrimental, the role of fusion proteins in this context remains uncertain.

**Objectives:**

To investigate whether Opa1 (protein involved in mitochondrial inner-membrane fusion) deficiency affects I/R injuries.

**Methods and Results:**

We examined mice exhibiting *Opa1*^delTTAG^ mutations (Opa1^+/-^), showing 70% Opa1 protein expression in the myocardium as compared to their wild-type (WT) littermates. Cardiac left-ventricular systolic function assessed by means of echocardiography was observed to be similar in 3-month-old WT and Opa1^+/-^ mice. After subjection to I/R, infarct size was significantly greater in Opa1^+/-^ than in WTs both *in vivo* (43.2±4.1% *vs*. 28.4±3.5%, respectively; p<0.01) and *ex vivo* (71.1±3.2% *vs*. 59.6±8.5%, respectively; p<0.05). No difference was observed in the expression of other main fission/fusion protein, oxidative phosphorylation, apoptotic markers, or mitochondrial permeability transition pore (mPTP) function. Analysis of calcium transients in isolated ventricular cardiomyocytes demonstrated a lower sarcoplasmic reticulum Ca^2+^ uptake, whereas cytosolic Ca^2+^ removal from the Na^+^/Ca^2+^ exchanger (NCX) was increased, whilst SERCA2a, phospholamban, and NCX protein expression levels were unaffected in Opa1^+/-^ compared to WT mice. Simultaneous whole-cell patch-clamp recordings of mitochondrial Ca^2+^ movements and ventricular action potential (AP) showed impairment of dynamic mitochondrial Ca^2+^ uptake and a marked increase in the AP late repolarization phase in conjunction with greater occurrence of arrhythmia in Opa1^+/-^ mice.

**Conclusion:**

Opa1 deficiency was associated with increased sensitivity to I/R, imbalance in dynamic mitochondrial Ca^2+^ uptake, and subsequent increase in NCX activity.

## Background

Mitochondria are double-membrane organelles that are essential to the life of eukaryotic cells, due to their special role at the crossroads of the survival and apoptotic pathways, and constitute the primary hosts of ATP production. This is particularly true in relation to the heart, which requires high rates of energy conversion, with mitochondria occupying around 30% of total cardiomyocyte volume [1].

Far removed from the traditional concept of static organelles, mitochondria have been revealed to be highly dynamic structures, altering their inner and outer membranes by means of fission and fusion mechanisms through the action of proteins located on inner and outer membranes [2]. The fission process, leading to mitochondrial fragmentation, is mediated by the cytosolic dynamin-related protein 1 (Drp1) and outer-membrane human fission factor 1 (Fis1). On the contrary, mitochondrial fusion is mediated by the action of outer-membrane mitofusins (Mfn 1 & 2) and inner-membrane optic atrophy factor 1 (Opa1). In line with others, our research team found that *OPA1* mutations were typically responsible for dominant optic atrophy (DOA, MIM#165500), an inherited disease affecting retinal ganglion cells while altering optic nerve integrity [3,4], in addition to a large spectrum of neurological syndromes [5].

Recent studies suggest involvement of mitochondrial dynamics in cardiovascular diseases, particularly in ischemia/reperfusion (I/R) injury, and infer potential protection means through modulation of fusion and fission protein expression [1]. Excessive fission has thus been associated with I/R injury and cell apoptosis [6]. Whilst some works suggest that inhibition of excessive mitochondrial fission through Fis1 and Drp1 modulation may be cardioprotective [7–9], the effects induced by modulation of the fusion proteins Mfn-2 and Opa1 were subject to contention. In fact, a fusion deficit induced through Mfn-2 deficiency had been unexpectedly linked to improved death protection and recovery after coronary artery ligation in an *in vivo* murine model [10]. Moreover, initial experiments conducted in Opa1-deficient models reported inconsistent results [11–13]. It is important to note that cardiomyocytes from Opa1-deficient mice models were more sensitive to simulated I/R [12], but it remains unknown whether this deficiency is of significance *in vivo* in the context of I/R.

The objective of this study was thus to investigate whether Opa1 deficiency would influence cardiac I/R injury *in vivo*. In order to respond to this question, we studied heterozygous mice carrying the most recurrent Opa1^delTTAG^ mutation, found in patients with dominant optic atrophy [14].

## Methods

### Opa1^delTTAG^ mice and breeding

As previously described, our group generated a knock-in *Opa1* mouse model carrying the recurrent Opa1^delTTAG^ mutation [15]. Briefly, C57Bl6/J mice were genetically modified to obtain the Opa1^delTTAG^ heterozygous mutation (Opa1^+/-^) that is observed in 30% of patients affected by dominant optic atrophy [15]. Homozygous Opa1^*-/-*^ is lethal in the early stages of fetal development. Male Opa1^+/-^ mice and their counterpart wild-type (WT) controls exhibiting Opa1^+/+^ were fed and hydrated without any restrictions. They were held in the animal facility of the UMR INSERM 1083-CNRS 6214 in Angers, France. All experiments were performed in compliance with the European Union and French guiding principles on the protection of animals used for scientific purposes (EU Directive 2010/63/EU; French Decree no. 2013–118). The protocol was approved by the local ethics committee (Comité d’Ethique en Expérimentation Animale des Pays de la Loire) and by the national committee (MENESR, 2015101511544187/APAFIS 3723).

### Echocardiography

Anesthetized mice (ketamine 60mg/Kg, intraperitoneal) underwent transthoracic echocardiography (TTE) as previously described [16]. Left-ventricular end-diastolic diameter (LVEDD), fractional shortening (FS), and heart rate were determined using 2D M-mode echocardiography. At the time of sacrifice, heart weight was measured and correlated to body weight (HW/BW).

### Blood pressure assessment

Blood pressure was measured by means of tail-cuff plethysmography (Visitech BP2000 System) in awake mice. For each mouse, the mean systolic blood pressure was averaged from 15 measurements recorded for 15 minutes every day for five consecutive days.

### Cardiomyocytes isolation and calcium transients

The mice’s hearts were subjected to enzymatic digestion (Liberase TH, Roche^®^) as previously described [17,18]. Immediately after excision and cannulation, hearts were retrogradely perfused at 37°C in a free-calcium perfusion buffer containing 5.5mM glucose, 113mM NaCl, 4.7mM KCl, 0.6mM KH_2_PO_4_, 0.6mM Na_2_HPO_4_, 1.2mM MgSO_4_, 12mM NaHCO_3_, 10mM KHCO_3_, 10mM HEPES, and 30mM taurine; pH = 7.4. The hearts were then perfused with the same buffer containing 0.1mg/mL Liberase TH (Roche^®^, France) for 5 to 10 minutes in order to achieve complete digestion. The left ventricles were then mechanically dissociated in the same solution, enzyme-free and containing 10mM of butanedione monoxime (BDM) (10% SVF), in order to stop enzymatic activity. Cells were transferred to a BDM-free buffer (5% SVF), and calcium concentration was progressively raised to a final concentration of 1mM.

Isolated cardiomyocytes were loaded with fluo-4 AM (4μM for 20 min, Molecular Probes, Eugene, Oregon, USA) and then placed on the stage of an inverted confocal microscope (LSM510 Meta Zeiss, Carl Zeiss, Jena, Germany) equipped with a 63x water-immersion objective (NA: 1.2). Cells were field-stimulated at 1Hz in a tyrode solution containing: 135mM NaCl, 4mM KCl, 1.8mM CaCl_2_, 1mM MgCl_2_, and 2mM HEPES (NaOH-adjusted pH of 7.4). Triggered calcium transients were recorded in line-scan mode (1.5ms/line, 3,000 lines/cell) along the longitudinal axis of the cell. Fluo-4 was excited at 488nm, and emitted light was collected using a 505nm-long pass filter. The laser intensity employed (<10% of maximum) had no noticeable detrimental effect on the fluorescent signal or on cell function over the course of the experiment.

Analysis was performed using the software ImageJ (NIH, Bethesda, Maryland, USA). In order to enable comparison between cells, the change in fluorescence (Δ*F*) was divided by the fluorescence detected immediately prior to the 1Hz stimulation pulse (*F0*). For assessment of sarcoplasmic reticulum (SR) Ca^2+^ content, cardiomyocytes were paced 1-2min at 1Hz to reach steady-state Ca^2+^ transients and SR Ca^2+^ load. Stimulation was subsequently stopped, and caffeine (10mM) applied to empty the SR.

The course of field-stimulated Ca^2+^ transients and caffeine-induced SR Ca^2+^ release was assessed by analyzing the maximum amplitude and time constant (*τ*) of the exponential part of the late decay phase. The time-to-peak normalized to the peak amplitude was also determined for stimulated Ca^2+^ transients.

### Mitochondrial calcium measurement and ventricular action potential

Mitochondrial Ca^2+^ movements and ventricular action potential (AP) were simultaneously recorded by means of a whole-cell patch-clamp technique, as previously described [18,19]. Briefly, after loading with Rhod-2 AM (5μM for 40 min at 37°C, TEFLabs, Austin, Texas, USA), the left-ventricular cardiomyocytes were placed on the stage of an inverted confocal microscope (LSM510 Meta Zeiss, 63x objective, NA: 1.2) and superfused with a standard tyrode solution containing 135mM NaCl, 4mM KCl, 1.8mM CaCl_2_, 1mM MgCl_2_, 2mM HEPES, and 10mM glucose at an NaOH-adjusted pH of 7.4. Subsequently, isolated cardiomyocytes were whole-cell-patch-clamped using an Axopatch 200B (Molecular Devices, Sunnyvale, California, USA), and patch pipettes (2-3MΩ) were filled with an internal solution containing 130mM KCl, 25mM HEPES, 3mM MgATP, 0.4mM NaGTP, 5mM NaCl, and 0.5mM EGTA at a KOH-adjusted pH of 7.2. Such an approach allows the dialysis of cytosolic rhod-2 [18,19]. APs were elicited using a current clamp by means of 0.2ms current injections of suprathreshold intensity and stimulated routinely at 1Hz as previously described [20]. Shifts in dye fluorescence were recorded in line-scan mode (1.54ms/line) along the short axis of the cell. As regards fluo-4 signals, confocal images were analyzed using the software ImageJ, and the change in fluorescence (Δ*F*) was divided by the fluorescence detected immediately prior to the 1Hz stimulation pulse (*F0*). APs were recorded and analyzed using the software program Pclamp 10 (Axon Instruments). AP durations were measured at 20%, 30%, 50%, 90%, and 95% of the repolarization phase.

### Mitochondrial morphology and function

#### Electron microscopy

Hearts from 3-month old mice were fixed by a retrogradely perfusion with 2% glutaraldehyde in cacodylate buffer (100mM sodium cacodylate and 2 mM MgCl_2_; pH 7.3). Then, left-ventricular papillary muscles were isolated and post-fixed as previously described [21]. Ultra-thin longitudinal sections were cut, and examined using an electron microscope. 20,000x-magnified images were used in the software ImageJ in order to assess the number and area of mitochondria. A mean of 20 fields were analyzed per mouse.

#### Mitochondrial respiration

Oxidative phosphorylation was determined as previously described [22]. The hearts were excised, and mitochondria isolated. Complex activity was assessed using 350μg of mitochondrial proteins. Complex I was determined by adding 5mM Glutamate/Malate/Puryvate (GMP), complex II by adding rotenone in conjunction with succinate, and complex IV by adding antimycine with PMDP ascorbate.

#### Mitochondrial permeability transition pore experiments

Mitochondrial permeability transition pore (mPTP) opening was assessed as previously described [23]. Calcium retention capacity (CRC) measurement using 250μg of mitochondrial proteins was performed at baseline and after the adjunction of cyclosporine A (1μM) in Opa1^+/-^ and WT mice.

### I/R experiments

#### Ex vivo I/R

Opa1^+/-^ and WT mice were anesthetized by means of an intraperitoneal injection of sodium pentobarbital (100mg/kg, Ceva Santé Animal^®^, Libourne, France). Heparin (1,000 IU/kg, Heparine Choay^®^) was also administered to prevent intracoronary clot formation. The heart was rapidly excised and immediately immersed in ice-cold modified Krebs-Henseleit buffer containing: 118.5mmol/l NaCl; 4.7mmol/l KCl; 25mmol/l NaHCO_3_; 1.2mmol/l MgSO_4_; 1.2mmol/l KH_2_PO_4_; 1.8mmol/l CaCl_2_; and 11mmol/l glucose (pH 7.4). The heart was mounted on a Langendorff-perfusion apparatus (ADInstruments, Dunedin, New Zealand) and retrogradely perfused through the aorta with non-recirculating buffer saturated with 95% O_2_ and 5% CO_2_ at 37°C. The heart was maintained in a thermostatic chamber at 37°C. Perfusion was maintained at a constant pressure of 80mmHg. After a 20-minute stabilization period, the hearts were subjected to 30 minutes of global ischemia and 2 hours of reperfusion.

#### In vivo I/R

Opa1^+/-^ and WT mice were anesthetized by means of intraperitoneal sodium pentobarbital injections (90mg/kg; Ceva Santé Animale) prior to endotracheal intubation and mechanical ventilation using MiniVent 845^®^ (Hugo-Sachs Elektronik—Harvard Apparatus GmbH, March, Germany). The mice received an injection of heparin (1,000 IU/kg, Heparine Choay^®^, Sanofi, Gentilly, France) prior to thoracotomy. Temperatures were monitored during the procedure and strictly maintained at 36.5° to 38°C. The thorax was opened via a left lateral thoracotomy, and the pericardium removed. Coronary ligation was performed on the left coronary artery using a monofilament (PROLENE 7.0^®^, Ethicon, LLC, Cincinnati, Ohio, USA), placed in a polyethylene tube to create a reversible snare, as previously described [24]. Ischemia was induced by clamping the tube and confirmed through observations of dyskinesia and cyanosis of the myocardial region below the suture. It was maintained for 45 minutes, followed by 120 minutes of reperfusion by loosening the snare.

#### Infarct size assessment

For *in vivo* experiments, coronary ligation was repeated at the end of the reperfusion period, and 400μl of Evans Blue (Sigma^®^) were injected through the left-ventricular apex, coloring the perfused (non-ischemic) myocardium blue. The area-at-risk (AAR) was then identified as the non-blue myocardium area and expressed as a percentage of left-ventricular area (LV). After heart extraction, the left ventricle was cut into six to seven slices before staining it with 2,3,5-triphenyltetrazolium chloride (TTC). The slices were subsequently submerged in 4% paraformaldehyde for 10 minutes, thereby delimiting the viable myocardium in red and area of necrosis (AN) in white. Quantification (planimetry) was performed using the software ImageJ 1.47. Infarct size was expressed as a percentage of the AAR (AN/AAR).

For isolated perfused hearts, infarct size was calculated after TTC staining and expressed as a percentage of the LV (AN/LV).

### Analysis of apoptosis

TUNEL staining was carried out on thin slices of heart tissue from AAR fixed in 4% paraformaldehyde, using the DeadEnd^™^ Fluorimetric TUNEL System (Promega^®^, Fitchburg, Wisconsin, USA), whilst adhering to manufacturers’ instructions. Propidium iodide (red) and fluorescein (green) were used to color total and apoptotic nuclei, respectively. Slides were observed using a confocal fluorescence microscope. Caspase-3 activity was determined using the Caspase 3 Colorimetric Assay Kit (Abcam^®^ plc, Cambridge, UK), thereby adhering to manufacturer’s instructions. Absorbance of ρ-nitroaniline was determined via spectrophotometry at 405nm and employed as an indirect indicator of the amount of substrate cleaved by caspase-3.

### Western blot analysis

Mitochondrial fission (Drp1, Fis1) and fusion protein (Opa1, Mfn2) expression was assessed at basal condition and after the *in vivo* I/R procedure by western blot analysis as previously described [25]. 40 μg of total proteins was separated by SDS-PAGE and transferred to a nitrocellulose or PVDF membrane. Primary antibody incubation was extended at night at 4°C for Opa1 (BD Transduction Laboratories^®^; 1/1000), Mfn2 (Sigma-Aldrich^®^; 1/2000), Fis1 (Santa Cruz^®^; 1/500), Drp1 (BD Transduction Laboratories^®^; 1/1000), Bax (Cell Signalling^®^; 1/1000), SERCA2a, NCX (Abcam; 1/1000), PLB, p-PLBser16 (Badrilla, 1/500, 1/2500) and Bcl-2 (BD Transduction Laboratories^®^; 1/1000). Membranes were incubated with appropriate secondary antibody conjugated to horseradish peroxidase (Santa Cruz Biotechnology). GAPDH expression was used as a loading control. The blots were developed using the enhanced chemiluminescence method. Semi-quantification of band intensity was performed using the software ImageJ.

### Statistical analysis

Statistical analyses were performed using the software SPSS Version 15.0 for Windows (SPSS Inc., Chicago, Illinois, USA). Results were expressed as mean±standard error of the mean (SEM). After testing linear variable distribution, group differences were assessed using ANOVA, followed by Student’s *t*-test. A non-parametric Mann-Whitney test was employed when appropriate. A p-value <0.05 was considered statistically significant.

## Results

### Cardiac structure and function in Opa1^+/-^ mice

**Fig 1:**
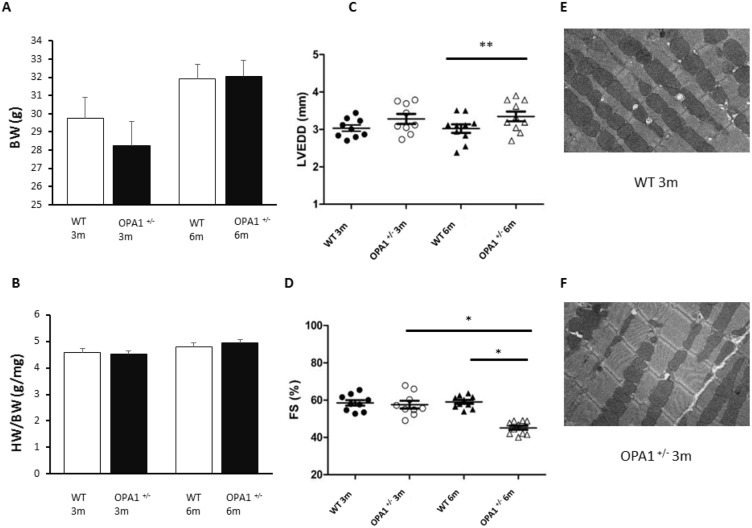
Cardiac morphology and function. BW (**A**) and HW/BW ratio (**B**) in 3- and 6-month-old Opa1^+/-^ (n = 11 and 12 respectively) and WT (n = 6 and 15 respectively) mice. **C, D**: Echocardiography parameters in 3- and 6-month-old mice,(n = 9/group at 3 months and 10/group at 6 months) whereby **C** represents left-ventricular end-diastolic diameter (LVEDD), and **D** depicts fractional shortening (FS). **E, F**: Examples of electron microscopy images at 12,000x magnification in 3-month-old WT (**E**) and Opa^+/-^ (**F**) mice. Values are mean ± SEM. * *p<0*.*05* and ** *p<0*.*01*. Non-parametric Mann-Whitney test (HW/BW) and t-test (BW, LVEDD and FS) according to distribution.

We characterized cardiac function by means of echocardiography in 3- and 6-month-old Opa1^+/-^ and WT mice. At 3 months of age, BW, HW, were not significantly different between Opa1^+/-^ (n = 11) and WT (n = 6) mice, as well as left-ventricular size and function (n = 9 per group) (Fig 1A–1D). At 6 months of age, Opa1^+/-^ mice exhibited an alteration of left-ventricular systolic function (FS = 45±1% in Opa1^+/-^ *vs*. 59±1% in WT [n = 10 per group], p<0.01), associated with significant dilation of the left ventricle (LVEDD = 3.35±0.13mm in Opa1^+/-^ *vs*. 2.97±0.1mm in WT group, p<0.05). As at 3 months, no statistical difference in BW and indexed HW was observed between Opa1^+/-^ and WT groups at 6 months. It is important to note that heart rates recorded at the time of echocardiography did not differ significantly between the groups at the same age (542±17bpm in WT and 548±20bpm in Opa1^+/-^ at 3 months, and 580±15bpm in WT and 545±20bpm in Opa1^+/-^ at 6 months). Systolic blood pressure likewise displayed no significant difference between groups at 3 months (120±2mmHg in WT and 118±1mmHg in Opa1^+/-^) and 6 months (120±1mmHg in WT and 122±2mmHg in Opa1^+/-^; n = 12/group).

Given that we previously reported aberrant structural conformation of myofibrils with large punctuated mitochondria and sarcomere disorganization, with large zones of autophagic and mitophagic materials in 5-month-old Opa1^+/-^ [15], we performed electron microscopy analysis in 3-month-old mice (n = 6/group). No difference was observed in structural conformation, nor the number and area of mitochondria between WT and Opa1^+/-^ mice (Fig 1E and 1F).

### Response of Opa1^+/-^ mice to I/R

In order to investigate whether a decreased expression of the fusion protein Opa1 affects I/R sensitivity, the following experiments were performed on 3-month-old mice exhibiting preserved cardiac morphological and functional parameters.

**Fig 2:**
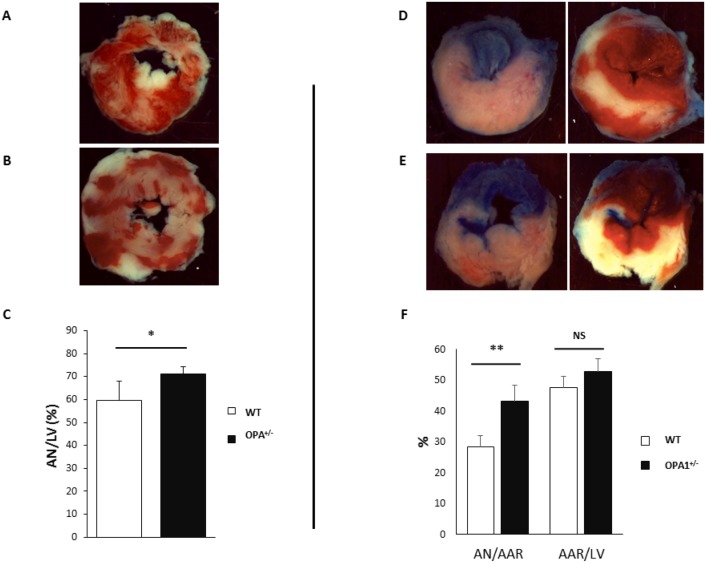
Infarct size. **A, B**: Examples of left-ventricular sections with TTC staining after 30 minutes of ischemia and 2 hours of reperfusion *ex vivo* in WT (**A**) and Opa1^+/-^ (**B**) mice, and histograms showing AN as a percentage of total LV in WT (n = 11) and OPA1^+/-^ (n = 12) mice (**C**). **D, E**: Examples of left-ventricular sections with TTC-staining after 45 minutes of ischemia and 2 hours of reperfusion *in vivo* in WT (**D**) and OPA1^+/-^ (**E**) mice. On the left side, images before TTC staining showing Evans blue coloration of the perfused myocardium and AAR as the non-blue area. On the right side, images after TTC staining showing AN in white. **F**: Histograms showing AN as a percentage of AAR and AAR as a percentage of total LV area in Opa1^+/-^ and WT mice (n = 8/group). Values are mean ± SEM. * *p<0*.05 and ** p<0.*01*. Non-parametric Mann-Whitney test (AN/AAR) and t-test (AAR/LV).

First, hearts from 11 WT and 12 Opa1^+/-^ mice were subjected to 30 minutes of *ex vivo* global ischemia and 2 hours of reperfusion. As shown in Fig 2A–2C, infarct size was significantly greater in the Opa1^+/-^ group as compared to the WT group (AN/LV = 71.1±3.2% *vs*. 59.6±8.5%, respectively; p<0.05). Similarly, Opa1^+/-^ mice displayed significantly greater infarct size than the control group when subjected *in vivo* to coronary artery ligation for 45 min, followed by 2 hours of reperfusion (AN/AAR = 43.2±4.1% in OPA1^+/-^ *vs*. 28.4±3.5% in WT, n = 8/group, p<0.01), whilst AAR/LV did not differ between groups (Fig 2D–2F). Thus, decreased Opa1 expression increased susceptibility to myocardial I/R.

### Evaluation of mitochondrial physiology

**Fig 3:**
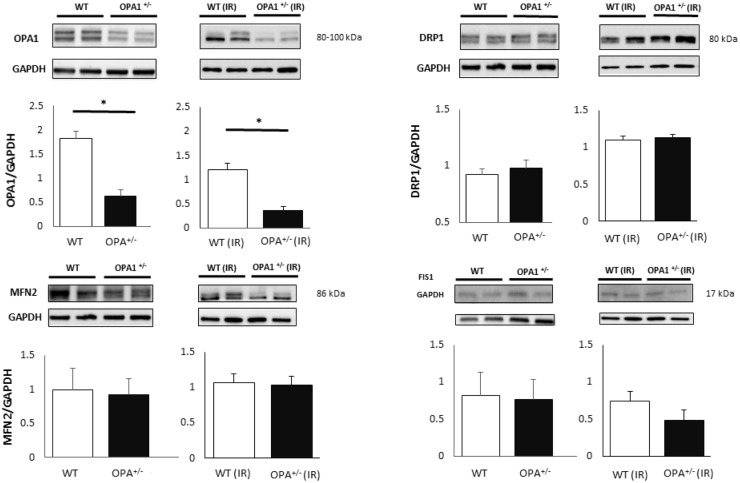
Baseline and post-I/R fission and fusion protein expression. Opa1, Mfn2, Drp1, and Fis1 expression assessed by means of western blotting at baseline (n = 6/group) and after I/R (n = 3-6/group). GAPDH was used as a loading control. Values are mean ± SEM. * p<0.01.

**Fig 4:**
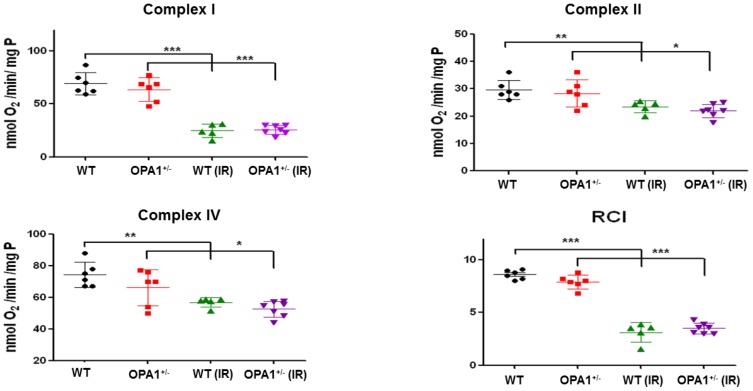
Baseline and post-I/R oxidative phosphorylation. Complex I, II, and IV oxygen consumption were measured in WT and Opa1^+/-^ mouse hearts at baseline and after I/R (n = 6/group). The I/R protocol consisted of 45 minutes of ischemia and 2 hours of reperfusion. The ratio [state 3 rate]: [state 4 rate] is represented by means of the respiratory control index (RCI) in both groups at baseline and after I/R. Values are mean ± SEM. *p<0.05, **p<0.01, ***p<0.001.

**Fig 5:**
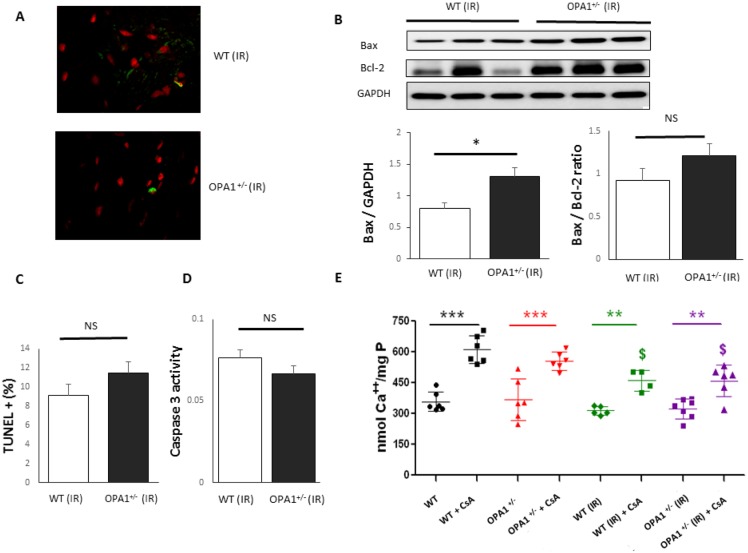
Apoptosis assessment and sensitivity to mPTP. **A**: Examples of TUNEL images of left-ventricular sections in confocal microscopy (70x magnification) after I/R. Cell nuclei are stained in red (propidium iodide, wavelength = 620 nm). TUNEL-positive nuclei appear in green (fluorescein, wavelength = 460 nm). **B**: Bax and Bcl-2 protein expressions (n = 4-6/group). **C**: Percentage of TUNEL-positive nuclei compared to total nuclei (n = 8 in Opa1^+/-^ and 7 in WT). **D**: Caspase 3 activity amongst Opa1^+/-^ (n = 7) and WT (n = 7) groups. **E**: mPTP opening sensitivity at baseline and after I/R (n = 6/group). Required calcium overload for mPTP opening in WT and Opa1^+/-^ groups at baseline, after I/R, with or without use of cyclosporine analog (CsA). The I/R protocol included 45 minutes of ischemia and 2 hours of reperfusion. Values are mean ± SEM. *p<0.05, **p<0.01, ***p<0.001. ^$^*p*< 0.05 compared to the same group at baseline + CsA. Non-parametric Mann-Whitney test (TUNEL and Bax/Bcl2 ratio) and t-test (Bax expression and caspase 3 activity).

To gain insight into the cellular mechanisms responsible for I/R susceptibility, we assessed mitochondrial parameters. First, we assessed whether decreased Opa1 expression affects the amounts of the other proteins involved in mitochondrial dynamics. Myocardial expression of Opa1, Mfn2 fusion and Drp1, Fis1 fission proteins were assessed in 3-month-old mice. Opa1 expression was significantly lower in Opa1^+/-^ mice (p<0.01), whilst expression of Drp1, Fis1, and Mfn2 was similar between the WT (n = 6) and Opa1^+/-^ (n = 6) groups (Fig 3). Similar results were obtained after 45 minutes of ischemia and 2 hours of reperfusion. We further characterized mitochondrial respiration in Opa1^+/-^ and WT mice, by assessing the respirations of complex I, II and IV in baseline conditions and state 3 / state 4 respiration ratio, without evidencing significant difference (n = 6/group). After 45 minutes of ischemia and 2 hours of reperfusion, oxidative phosphorylation was clearly reduced in both groups, with no significant difference between Opa1^+/-^ and WT mice (Fig 4). In addition, we evaluated the parameters related to apoptosis, and found that the expression of the pro-apoptotic Bax after I/R was significantly higher in the *Opa1*^+/-^ group (n = 4) compared to the WT group (n = 6) (p<0.05) (Fig 5A–5D), although Bcl-2 expression and Bax/Bcl-2 ratio were not different between groups. Moreover, the number of apoptotic TUNEL-positive nuclei did not differ between Opa1^+/-^ and WT groups (respectively 11.5±3.5%, n = 8 *vs*. 9.1±2.7%, n = 7; p = 0.56) nor the caspase-3 activity (n = 7/group; p = 0.21).

### mPTP function

Finally, we assessed the opening of the mPTP. Under baseline conditions, calcium retention capacity was not significantly different in the Opa1^+/-^ group than in the WT group (Fig 5E). Administration of cyclosporine A, a selective mPTP inhibitor, significantly increased calcium retention, but to similar degrees in both groups. After 45 minutes of ischemia and 2 hours of reperfusion, Opa1^+/-^ mice displayed similar calcium retention capacities to WT mice. Moreover, there was no significant difference in baseline and post-I/R calcium retention capacity. When compared to baseline, the post-I/R effect of cyclosporine A adjunction was reduced.

### Calcium transients

**Fig 6:**
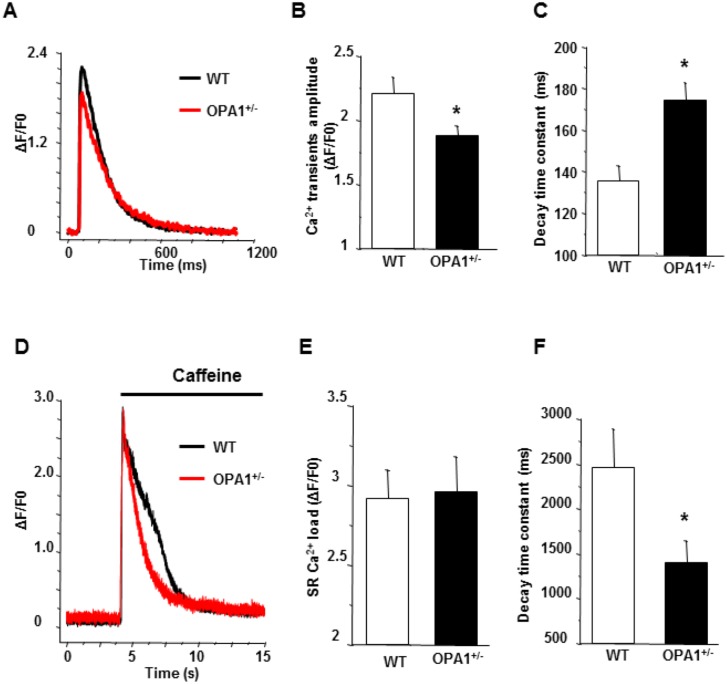
Calcium transients and SR calcium load in Opa1^+/-^ isolated left-ventricular cardiomyocytes. **A**: Typical calcium transients recorded under field stimulation at 1Hz in WT (black) and Opa1^+/-^ (red) isolated left-ventricular cardiomyocytes using fluo-4 calcium dye. **B**: Mean values of peak calcium transients (WT, n = 32 cells and 4 animals, *vs*. Opa1^+/-^, n = 43 cells and 5 animals; *p<0.05). **C**: Mean values of decay time constant of steady-state Ca^2+^ transients (WT, n = 32 cells and 4 animals, *vs*. Opa1^+/-^, n = 43 cells and 5 animals; *p<0.05). **D**: Typical example of a caffeine-induced SR Ca^2+^ release in WT (black) and Opa1^+/-^ (red) left-ventricular cardiomyocytes using fluo-4 calcium dye. **E**: Mean values of maximum amplitude of caffeine-induced SR Ca^2+^ release, indicative of SR Ca^2+^ load (WT, n = 7 cells and 4 animals, *vs*. Opa1^+/-^, n = 11 cells and 5 animals; p>0.05). **F**: Mean values of decay time constant of caffeine-induced SR Ca^2+^ release, indicative of cytosolic Ca^2+^ extrusion (WT, n = 7 cells and 4 animals, *vs*. Opa1^+/-^, n = 11 cells and 5 animals; *p<0.05). Statistical test was t-test.

We subsequently compared calcium transients in isolated cardiomyocytes stimulated at 1Hz. In Opa1^+/-^ left-ventricular cells, the amplitude of calcium transients was significantly lower and exhibited a slower decay time constant compared to WT cardiomyocytes (Fig 6A–6C). This indicates that SR calcium uptake is affected in Opa1^+/-^ cells. Similarly, the extend of caffeine-induced SR calcium release was comparable in WT and Opa1^+/-^ myocytes. That said, the decay time constant, which depends primarily upon sarcolemmal Na^+^/Ca^2+^ exchanger (NCX) activity, proved to be much faster in Opa1^+/-^ myocytes (Fig 6D–6F). Overall, these results suggest that, in Opa1^+/-^, SR Ca^2+^ uptake was decreased, whereas cytosolic Ca^2+^ removal due to NCX increased. It is important to note that myocardial expressions of SERCA2a, total and phosphorylated phospholamban, and NCX proteins were not significantly different between WT (n = 6) and Opa1^+/-^ (n = 4) groups in 3-month-old mice.

**Fig 7:**
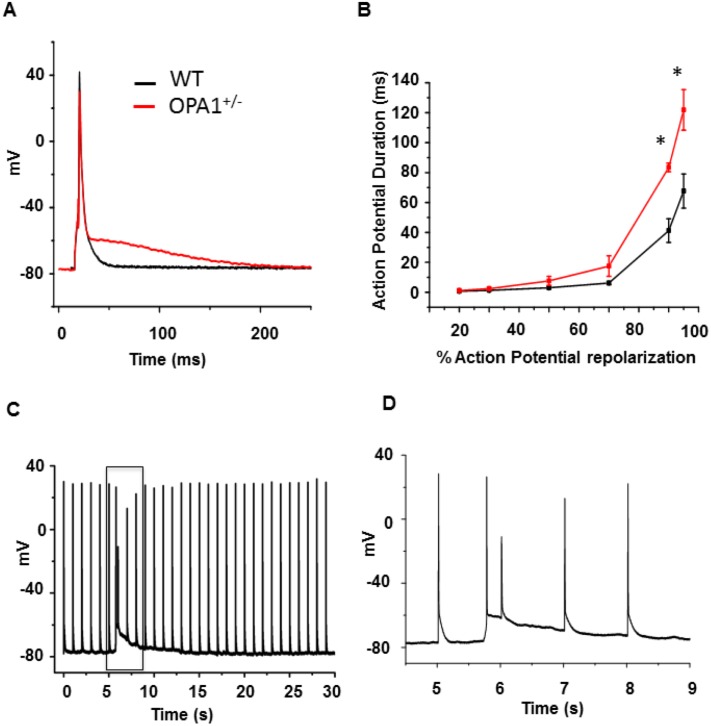
Opa1^+/-^ left-ventricular cardiomyocyte AP. **A**: Typical AP recorded using whole-cell patch-clamp technique with current clamp at 1Hz in WT (black) and Opa1^+/-^ (red) isolated left-ventricular cardiomyocytes. **B**: Mean values of AP duration at different percentages of AP repolarization (WT: n = 12 cells and 3 animals *vs*. Opa1^+/-^: n = 12 cells and 4 animals; *p<0.05). **C**, **D**: Frame **C** displays 30 seconds of continuous AP recording at a pacing rate of 1Hz early after depolarization, whilst frame **D** shows a magnified view of the early post-depolarization period. Note that 6/12 Opa1^+/-^ cells present such arrhythmic events, whereas WT myocytes underwent no early subsequent depolarization. Statistical test was t-test.

**Fig 8:**
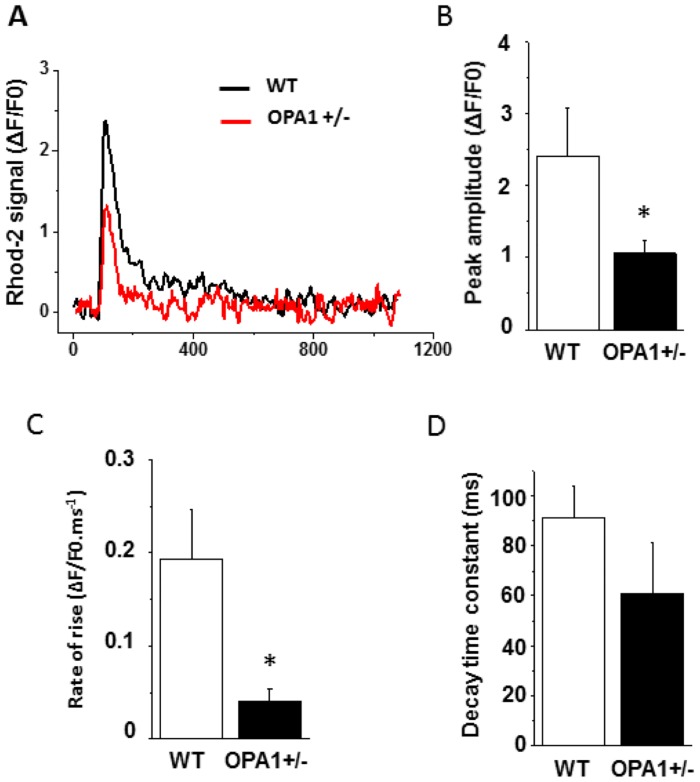
Dynamic mitochondrial Ca^2+^ movements during APs recorded using Rhod-2 in conjunction with a whole-cell patch-clamp technique. **A**: Typical rhod-2 signal during a steady-state AP recorded at 1Hz in WT (black) and Opa1^+/-^ (red) isolated left-ventricular cardiomyocytes. **B**: Mean values of peak rhod-2 signal. **C**: Rate of rise. **D**: Decay time constant in WT (n = 12 cells and 3 animals) and Opa1^+/-^ cells (n = 12 cells and 4 animals; *p<0.05). Statistical test was t-test.

We subsequently determined whether mitochondrial Ca^2+^ movements and AP durations were affected in Opa1^+/-^ ventricular cardiomyocytes. To this end, we simultaneously recorded rhod-2 signals and AP using a whole-cell patch-clamp technique. As shown in Fig 7, and in accordance with increased Ca^2+^ extrusion through NCX, AP durations in Opa1^+/-^ cardiomyocytes were significantly increased during the late phase of repolarization. Furthermore, 50% of Opa1^+/-^ cardiomyocytes were triggered soon after depolarization (6/12 cells), whereas no such arrhythmia was observed in WT cardiomyocytes (Fig 7). On the other hand, mitochondrial Ca^2+^ uptake during steady-state AP was lower and exhibited a slower rate of increase. The mitochondrial Ca^2+^ decay time constant was not significantly affected in Opa1^+/-^ cardiomyocytes compared to the WT group (Fig 8). Altogether, these results indicate that in Opa1^+/-^ cardiomyocytes, mitochondrial Ca^2+^ uptake is impaired, whereas the late repolarization phase of APs is markedly increased with an enhanced arrhythmia triggering.

## Discussion

The susceptibility of the myocardium to develop I/R injury is highly related to the mitochondrial functions [26]. Indeed, during ischemia, cardiac mitochondria are subjected to hypoxia, calcium overload, low pH, and ATP depletion. Reperfusion abruptly subjects mitochondria to a rapid pH recovery, oxidative stress, restoration of mitochondrial membrane potential, and calcium overload, all of which inducing mPTP opening [27]. mPTP opening at reperfusion onset is a primary trigger of cardiomyocyte death [28]. Of note, modulation of mitochondrial dynamics may affect susceptibility to I/R injury by preventing mitochondrial dysfunction and mPTP inhibition [6]. Whilst excessive fission has been associated with I/R injury and cell death, it was unclear whether loss of the fusion protein Opa1 likewise contributes to the occurrence of I/R injury *in vivo*. Our study demonstrated that Opa1 deficiency is associated with increased sensitivity to I/R in mice exhibiting the Opa1^delTTAG^ mutation, both *ex vivo* and *in vivo*.

Several studies have reported mitochondrial fragmentation in cardiac cells subjected to simulated I/R [6,11,29], a phenomenon associated with a reduction in Opa1 protein expression [11]. However, most of these studies were performed *in vitro*. Ong et al. have shown that suppression of fission and stimulation of fusion by Drp1 downregulation, expression of dominant-negative Drp1, treatment with mdivi-1, or overexpression of Mfn1 or Mfn2 in HL-1 cardiac cell, prevented mPTP opening and reduced cell death after I/R [6].

In order to investigate *in vivo* the role of Opa1 deficiency in cardiac I/R injury, we studied our previously generated knock-in OPA1 mouse model [15]. This model carrying the recurrent Opa1^delTTAG^ heterozygous mutation affected one third of patients exhibiting dominant optic atrophy. The mouse displayed a multi-systemic poly-degenerative phenotype and presented a combination of visual failure, deafness, encephalomyopathy, peripheral neuropathy, ataxia, and cardiomyopathy [15]. Whilst cardiac muscles from these mice displayed aberrant structural conformation of myofibrils with large punctuated mitochondria but normal cristae organization and sarcomer disorganization, with large zones of autophagic and mitophagic materials at 5 months [15], we observed no difference with the WT littermates at 3 months of age, indicating age-related onset of cardiomyopathy.

In line with the age-related development of cardiac muscular dysfunction and mitochondrial structure abnormalities in this mouse model, we observed normal cardiac function at 3 months and moderate alteration of left-ventricular systolic function at 6 months. In another knock-in Opa1 model, Chen *et al*. reported similar, late-onset, cardiac dysfunction, associated with no significant cardiac functional or gross structural abnormalities at 3 months, but impaired systolic function at 12 months [12]. In our Knowledge, cardiac involvement in patients carrying OPA1 mutation has only been recently reported for the first time in two sisters harboring a homozygous OPA1 mutation leading to lethal encephalopathy and hypertrophic cardiomyopathy [30]. As we wished to explore the role of Opa1 deficiency prior to the occurrence of cardiac functional impairment, we opted to subject the mice to I/R at 3 months. At this age, Opa1 expression had decreased by 70% in the heart and, importantly, none of the other major fusion/fission proteins (Drp1, Fis1, and Mfn2) were affected.

Similarly to the data observed *in vitro* in other Opa1-deficient models, we recorded greater infarct size in Opa1-deficient mice subjected to I/R *in vivo*. Although acute I/R injury has been linked to mPTP opening at reperfusion onset following a period of ischemia [27], we observed no increased sensitivity to mPTP in Opa1-deficient mice either at baseline or after I/R. Furthermore, mitochondrial respiration was also not impaired in Opa1-deficient mice at baseline, and oxidative phosphorylation was lower both in OPA1-deficient mice and controls after I/R.

Calcium flux constitutes one of the major cellular factors in myocardial I/R injury [31,32]. As stated, Opa1^+/-^ ventricular cardiomyocytes displayed a reduced calcium transient amplitude with a slower decay time constant, whereas the rate of calcium transient increase remained unaffected compared to WT cardiomyocytes [12]. Furthermore, our data suggests enhanced cytosolic Ca^2+^ removal by means of the NCX, characterized by a faster decline of the caffeine-induced Ca^2+^ transients and a drastic increase in the late repolarization phase of the Aps [33,34]. Increased NCX activity is associated with greater occurrence of arrhythmias, such as delayed after depolarization (DADs) and/or early after depolarization (EADs) [35]. The involvement of NCX in DADs is well-established and is related to increased NCX-dependent Ca^2+^ extrusion during diastole, which generates an inward current and the resting membrane potential depolarization, whereas the NCX contribution to EADs genesis is more complex. Increased Ca^2+^ extrusion during the AP repolarization phase slows down AP repolarization, favoring l-type Ca2+ current reactivation and triggering of EADs [36]. Enhanced NCX activity and increased AP were not associated with an increased expression of NCX in Opa1^+/-^ or a shift in SR Ca^2+^ load and SERCA2a^2+^/PLB, but with a decrease in beat-to-beat mitochondrial Ca^2+^ uptake. Decreased dynamic mitochondrial Ca^2+^ uptake may thus destabilize Ca^2+^ flux equilibrium between SR Ca^2+^ uptake, NCX Ca^2+^ removal, and mitochondria in controlling cytosolic Ca^2+^ homeostasis during Aps [37]. Whilst no definitive explanation prevails as to why Opa1 deficiency is associated with increased susceptibility to cardiac I/R injury, it may be postulated that an imbalance in Ca^2+^ fluxes and the subsequent increase in NCX activity may contribute to the observed reperfusion injury. Indeed, increased NCX Ca^2+^ extrusion may raise steady-state intracellular Na^+^ levels during normoxia, which, during ischemia, may combine with the intracellular Na^+^ rise mediated by Na^+^/K^+^ ATPase inhibition and acidosis. Increased ischemic Na^+^ levels would thus raise Ca^2+^ influx via NCX reverse mode, thereby increasing cytosolic Ca^2+^ overload during ischemia and subsequent reperfusion injuries [35].

On the other hand, Varanita T *et al*. recently reported genetically mild Opa1 overexpression to be associated with reduced occurrence of cardiac injury in Langendorff-perfused hearts subjected to I/R [38]. It is worth noting that protection from I/R injury was not limited to the heart, as infarct size was likewise smaller in brains subjected to I/R. The commonly accepted conclusion is that fused mitochondria are more tolerant to ischemia and better adapt to factors causing mPTP opening at reperfusion onset. That said, a recent report has been the subject of contention regarding this concept. In a cardiac-specific Drp1 heterozygous knockout mouse, Ikeda *et al*. reported cardiac mitochondrial elongation associated with increased susceptibility to I/R [39]. Drp1 downregulation induced accumulation of damaged mitochondria and impaired mitochondrial autophagy. This data underscores that mitochondrial dynamics is an active process requiring a tight equilibrium between fusion and fission in order to maintain efficient cardiac cell functions, especially in critical situations such as I/R.

### Limits

We employed a knock-in *Opa1* mouse model carrying the recurrent Opa^delTTAG^ mutation in order to assess the role of mitochondrial dynamics in the development of I/R injury *in vivo* as well as *ex vivo*. As a consequence of this chronic Opa1 deficiency, several unsuspected compensation mechanisms may have occurred in addition to the increase in NCX activity.

Moreover, we observed no significant increase in apoptosis in Opa1-deficient mice as an underlying cause for their higher infarct size. Due to the dynamic nature of apoptosis, it could be postulated that we overlooked such an increase by assessing apoptosis at single point in time. It is worth noting that Chen *et al*. likewise did not find any marker of increased apoptosis activity in their OPA1-deficient model using TUNEL, histological, Bak1, bcl2, bcl2/1, Bnip3, bk, and bax analysis [12]. Finally, calcium regulation was investigated in normoxic cardiomyocytes. It would be of interest to obtain similar information after hypoxia/reoxygenation.

## Conclusion

Opa1 deficiency was associated with an increased sensitivity to I/R in mice with the Opa1^delTTAG^ mutation, both *ex vivo* and *in vivo*. The mechanism seems to be related to an imbalance in dynamic mitochondrial Ca^2+^ uptake and the subsequent increase in NCX activity.

## References

[1] Hall AR, Burke N, Dongworth RK, Hausenloy DJ. Mitochondrial fusion and fission proteins: novel therapeutic targets for combating cardiovascular disease. Br J Pharmacol 2014;171:1890–1906. 10.1111/bph.12516 24328763 PMC3976611

[2] Youle RJ, van der Bliek AM. Mitochondrial fission, fusion, and stress. Science 2012;337:1062–1065. 10.1126/science.1219855 22936770 PMC4762028

[3] Alexander C, Votruba M, Pesch UE, Thiselton DL, Mayer S, Moore A et al OPA1, encoding a dynamin-related GTPase, is mutated in autosomal dominant optic atrophy linked to chromosome 3q28. Nat Genet 2000;26:211–215. 10.1038/79944 11017080

[4] Delettre C, Lenaers G, Griffoin JM, Gigarel N, Lorenzo C, Belenguer P et al Nuclear gene OPA1, encoding a mitochondrial dynamin-related protein, is mutated in dominant optic atrophy. Nat Genet 2000;26:207–210. 10.1038/79936 11017079

[5] Chao de la Barca JM, Prunier-Mirebeau D, Amati-Bonneau P, Ferre M, Sarzi E, Bris C, et al OPA1-related disorders: Diversity of clinical expression, modes of inheritance and pathophysiology. Neurobiol Dis 2016;90:20–26. 10.1016/j.nbd.2015.08.015 26311407

[6] Ong SB, Subrayan S, Lim SY, Yellon DM, Davidson SM, Hausenloy DJ. Inhibiting mitochondrial fission protects the heart against ischemia/reperfusion injury. Circulation 2010;121:2012–2022. 10.1161/CIRCULATIONAHA.109.906610 20421521

[7] Disatnik MH, Ferreira JC, Campos JC, Gomes KS, Dourado PM, Qi X et al Acute inhibition of excessive mitochondrial fission after myocardial infarction prevents long-term cardiac dysfunction. J Am Heart Assoc 2013;2:e000461 10.1161/JAHA.113.000461 24103571 PMC3835263

[8] Sharp WW, Fang YH, Han M, Zhang HJ, Hong Z, Banathy A et al Dynamin-related protein 1 (Drp1)-mediated diastolic dysfunction in myocardial ischemia-reperfusion injury: therapeutic benefits of Drp1 inhibition to reduce mitochondrial fission. Faseb j 2014;28:316–326. 10.1096/fj.12-226225 24076965 PMC3868827

[9] Zepeda R, Kuzmicic J, Parra V, Troncoso R, Pennanen C, Riquelme JA et al Drp1 loss-of-function reduces cardiomyocyte oxygen dependence protecting the heart from ischemia-reperfusion injury. J Cardiovasc Pharmacol 2014;63:477–487. 10.1097/FJC.0000000000000071 24477044

[10] Papanicolaou KN, Khairallah RJ, Ngoh GA, Chikando A, Luptak I, O'Shea KM et al Mitofusin-2 maintains mitochondrial structure and contributes to stress-induced permeability transition in cardiac myocytes. Mol Cell Biol 2011;31:1309–1328. 10.1128/MCB.00911-10 21245373 PMC3067905

[11] Chen L, Gong Q, Stice JP, Knowlton AA. Mitochondrial OPA1, apoptosis, and heart failure. Cardiovasc Res 2009;84:91–99. 10.1093/cvr/cvp181 19493956 PMC2741347

[12] Chen L, Liu T, Tran A, Lu X, Tomilov AA, Davies V et al OPA1 mutation and late-onset cardiomyopathy: mitochondrial dysfunction and mtDNA instability. J Am Heart Assoc 2012;1:e003012 10.1161/JAHA.112.003012 23316298 PMC3541627

[13] Piquereau J, Caffin F, Novotova M, Prola A, Garnier A, Mateo P et al Down-regulation of OPA1 alters mouse mitochondrial morphology, PTP function, and cardiac adaptation to pressure overload. Cardiovasc Res 2012;94:408–417. 10.1093/cvr/cvs117 22406748 PMC3863708

[14] Ferre M, Caignard A, Milea D, Leruez S, Cassereau J, Chevrollier A et al Improved locus-specific database for OPA1 mutations allows inclusion of advanced clinical data. Hum Mutat 2015;36:20–25. 10.1002/humu.22703 25243597

[15] Sarzi E, Angebault C, Seveno M, Gueguen N, Chaix B, Bielicki G et al The human OPA1delTTAG mutation induces premature age-related systemic neurodegeneration in mouse. Brain 2012;135:3599–3613. 10.1093/brain/aws303 23250881

[16] Prunier F, Gaertner R, Louedec L, Michel JB, Mercadier JJ, Escoubet B. Doppler echocardiographic estimation of left ventricular end-diastolic pressure after MI in rats. Am J Physiol Heart Circ Physiol 2002;283:H346–352. 10.1152/ajpheart.01050.2001 12063308

[17] Fauconnier J, Lanner JT, Zhang SJ, Tavi P, Bruton JD, Katz A et al Insulin and inositol 1,4,5-trisphosphate trigger abnormal cytosolic Ca2+ transients and reveal mitochondrial Ca2+ handling defects in cardiomyocytes of ob/ob mice. Diabetes 2005;54:2375–2381. 10.2337/diabetes.54.8.2375 16046304

[18] Paillard M, Tubbs E, Thiebaut PA, Gomez L, Fauconnier J, Da Silva CC et al Depressing mitochondria-reticulum interactions protects cardiomyocytes from lethal hypoxia-reoxygenation injury. Circulation 2013;128:1555–1565. 10.1161/CIRCULATIONAHA.113.001225 23983249

[19] Dague E, Genet G, Lachaize V, Guilbeau-Frugier C, Fauconnier J, Mias C et al Atomic force and electron microscopic-based study of sarcolemmal surface of living cardiomyocytes unveils unexpected mitochondrial shift in heart failure. J Mol Cell Cardiol 2014;74:162–172. 10.1016/j.yjmcc.2014.05.006 24839910

[20] Fauconnier J, Bedut S, Le Guennec JY, Babuty D, Richard S. Ca2+ current-mediated regulation of action potential by pacing rate in rat ventricular myocytes. Cardiovasc Res 2003;57:670–680. 10.1016/S0008-6363(02)00731-9 12618229

[21] Wilding JR, Joubert F, de Araujo C, Fortin D, Novotova M, Veksler V et al Altered energy transfer from mitochondria to sarcoplasmic reticulum after cytoarchitectural perturbations in mice hearts. J Physiol 2006;575:191–200. 10.1113/jphysiol.2006.114116 16740607 PMC1819422

[22] Teixeira G, Chiari P, Fauconnier J, Abrial M, Couture-Lepetit E, Harisseh R et al Involvement of Cyclophilin D and Calcium in Isoflurane-induced Preconditioning. Anesthesiology 2015;123:1374–1384. 10.1097/ALN.0000000000000876 26460965

[23] Gharib A, De Paulis D, Li B, Augeul L, Couture-Lepetit E, Gomez L et al Opposite and tissue-specific effects of coenzyme Q2 on mPTP opening and ROS production between heart and liver mitochondria: role of complex I. J Mol Cell Cardiol 2012;52:1091–1095. 10.1016/j.yjmcc.2012.02.005 22387164

[24] Kalakech H, Tamareille S, Pons S, Godin-Ribuot D, Carmeliet P, Furber A et al Role of hypoxia inducible factor-1alpha in remote limb ischemic preconditioning. J Mol Cell Cardiol 2013;65:98–104. 10.1016/j.yjmcc.2013.10.001 24140799

[25] Tamareille S, Mateus V, Ghaboura N, Jeanneteau J, Croue A, Henrion D et al RISK and SAFE signaling pathway interactions in remote limb ischemic perconditioning in combination with local ischemic postconditioning. Basic Res Cardiol 2011;106:1329–1339. 10.1007/s00395-011-0210-z 21833651

[26] Heusch G. Molecular basis of cardioprotection: signal transduction in ischemic pre-, post-, and remote conditioning. Circ Res 2015;116:674–699. 10.1161/CIRCRESAHA.116.305348 25677517

[27] Gomez L, Li B, Mewton N, Sanchez I, Piot C, Elbaz M et al Inhibition of mitochondrial permeability transition pore opening: translation to patients. Cardiovasc Res 2009;83:226–233. 10.1093/cvr/cvp063 19221132

[28] Heusch G, Boengler K, Schulz R. Inhibition of mitochondrial permeability transition pore opening: the Holy Grail of cardioprotection. Basic Res Cardiol 2010;105:151–154. 10.1007/s00395-009-0080-9 20066536

[29] Brady NR, Hamacher-Brady A, Gottlieb RA. Proapoptotic BCL-2 family members and mitochondrial dysfunction during ischemia/reperfusion injury, a study employing cardiac HL-1 cells and GFP biosensors. Biochim Biophys Acta 2006;1757:667–678. 10.1016/j.bbabio.2006.04.011 16730326

[30] Spiegel R, Saada A, Flannery PJ, Burte F, Soiferman D, Khayat M et al Fatal infantile mitochondrial encephalomyopathy, hypertrophic cardiomyopathy and optic atrophy associated with a homozygous OPA1 mutation. J Med Genet 2016;53:127–131. 10.1136/jmedgenet-2015-103361 26561570 PMC4752660

[31] Fauconnier J, Roberge S, Saint N, Lacampagne A. Type 2 ryanodine receptor: a novel therapeutic target in myocardial ischemia/reperfusion. Pharmacol Ther 2013;138:323–332. 10.1016/j.pharmthera.2013.01.015 23384595

[32] Prunier F, Kawase Y, Gianni D, Scapin C, Danik SB, Ellinor PT et al Prevention of ventricular arrhythmias with sarcoplasmic reticulum Ca2+ ATPase pump overexpression in a porcine model of ischemia reperfusion. Circulation 2008;118:614–624. 10.1161/CIRCULATIONAHA.108.770883 18645052

[33] Bogeholz N, Pauls P, Bauer BK, Schulte JS, Dechering DG, Frommeyer G et al Suppression of Early and Late Afterdepolarizations by Heterozygous Knockout of the Na+/Ca2+ Exchanger in a Murine Model. Circ Arrhythm Electrophysiol 2015;8:1210–1218. 10.1161/CIRCEP.115.002927 26338832

[34] Yao A, Su Z, Nonaka A, Zubair I, Lu L, Philipson KD et al Effects of overexpression of the Na+-Ca2+ exchanger on [Ca2+]i transients in murine ventricular myocytes. Circ Res 1998;82:657–665. 10.1161/01.RES.82.6.657 9546374

[35] Pott C, Eckardt L, Goldhaber JI. Triple threat: the Na+/Ca2+ exchanger in the pathophysiology of cardiac arrhythmia, ischemia and heart failure. Curr Drug Targets 2011;12:737–747. 10.2174/138945011795378559 21291388 PMC4406235

[36] Qu Z, Xie LH, Olcese R, Karagueuzian HS, Chen PS, Garfinkel A et al Early afterdepolarizations in cardiac myocytes: beyond reduced repolarization reserve. Cardiovasc Res 2013;99:6–15. 10.1093/cvr/cvt104 23619423 PMC3687754

[37] Bers DM. Calcium fluxes involved in control of cardiac myocyte contraction. Circ Res 2000;87:275–281. 10.1161/01.RES.87.4.275 10948060

[38] Varanita T, Soriano ME, Romanello V, Zaglia T, Quintana-Cabrera R, Semenzato M et al The OPA1-dependent mitochondrial cristae remodeling pathway controls atrophic, apoptotic, and ischemic tissue damage. Cell Metab 2015;21:834–844. 10.1016/j.cmet.2015.05.007 26039448 PMC4457892

[39] Ikeda Y, Shirakabe A, Maejima Y, Zhai P, Sciarretta S, Toli J et al Endogenous Drp1 mediates mitochondrial autophagy and protects the heart against energy stress. Circ Res 2015;116:264–278. 10.1161/CIRCRESAHA.116.303356 25332205

